# What Caused Declines in Intertidal Invertebrate Populations around Fukushima Daiichi Nuclear Power Plant after the 2011 Great East Japan Earthquake, Tsunami, and Nuclear Disaster?

**DOI:** 10.3390/toxics10050214

**Published:** 2022-04-24

**Authors:** Toshihiro Horiguchi, Keita Kodama

**Affiliations:** Ecosystem Impact Research Section, Health and Environmental Risk Division, National Institute for Environmental Studies, 16-2 Onogawa, Tsukuba 305-8506, Japan; kodama.keita@nies.go.jp

**Keywords:** aquatic organisms, continuous sexual maturation, defaunation, intertidal invertebrates, ionizing radiation, nuclear disaster, population decline, population densities, population-level effects assessment, radionuclides

## Abstract

We discuss possible causal factors for the decline in intertidal invertebrate populations around Fukushima Daiichi Nuclear Power Plant (FDNPP) after the 2011 Great East Japan Earthquake and subsequent tsunami and nuclear disaster on the basis of existing knowledge about the effects of radionuclides and ionizing radiation on aquatic organisms. We found a gap between effects observed in the laboratory and those observed in natural aquatic environments, and discuss possible reasons why. Considering the complexity of the environment, we conclude that it is critical to evaluate the effects of ionizing radiation combined with other biotic and abiotic environmental factors, together with the life-history traits of the species examined, for realistic assessment of population-level effects. Finally, we present possible causal factors for strange or abnormal phenomena observed in intertidal biota near FDNPP, namely declines in population densities and number of species of invertebrates, delayed recovery from these declines, and continuous sexual maturation in the rock shell population.

## 1. Possible Impacts to Marine Organisms around Fukushima Daiichi Nuclear Power Plant after the 2011 Great East Japan Earthquake, Tsunami, and Nuclear Disaster

After the 2011 Great East Japan Earthquake, and the subsequent tsunami and disaster at the Fukushima Daiichi Nuclear Power Plant (FDNPP), Garnier-Laplace et al. [1] calculated dose rates from radionuclides in Fukushima’s most affected areas and suggested that more severe impacts were likely in the coastal ecosystem adjacent to FDNPP than in forest ecosystems. Maximum dose rates for ^131^I, ^134^Cs and ^137^Cs were estimated to range from 210 to 4600 mGy/d for marine birds, fish, mollusks, crustaceans and brown algae. Based on these high dose rates, marked reproductive effects (and even mortality in the most radiosensitive taxa) were implied in all marine wildlife groups in the vicinity of FDNPP. This was under the assumption that there were no additional releases of radionuclides into the sea after March 2011, and in the absence of any estimates of dose rates from other possible radionuclides (e.g., ^58^Co, ^95^Zr, ^99^Mo, ^99m^Tc, ^105^Ru, ^106^Ru, ^129m^Te, ^129^Te, ^132^Te, ^136^Cs, ^132^I, ^140^Ba, ^140^La) [1]. However, Vives i Batlle [2] estimated radiation doses to marine biota near FDNPP for the immediate aftermath of the accident and the subsequent period, using monitoring data that had been complemented by means of dynamic transfer modelling. Vives i Batlle [2] then suggested that earlier assessments using equilibrium transfer models overestimated the exposures immediately after the accident, whereas dynamic transfer modelling brought them in accordance with the doses calculated from monitored activity concentrations in the biota. From this, Vives i Batlle [2] concluded that marine biota near FDNPP did not seem to be at significant risk.

On the other hand, several strange phenomena (or possibly abnormal observations) have been reported in invertebrates in the intertidal zone near FDNPP, and in megabenthos (i.e., fishes, crustaceans, mollusks and echinoderms) in the coastal waters off Fukushima Prefecture. In 2011, 2012 and 2013, Horiguchi et al. [3] investigated the intertidal zones of eastern Japan to examine the ecological effects of the accident at FDNPP. The number of intertidal species decreased significantly with decreasing distance from FDNPP, and no rock shell (*Thais clavigera*, currently recognized as *Reishia clavigera*; Gastropoda, Neogastropoda, Muricidae) specimens were collected near FDNPP, from Hirono to Futaba Beach (a distance of approximately 30 km) in 2012. The collection of rock shell specimens at many other sites hit by the tsunami implies that the absence of rock shells around FDNPP in 2012 might have been caused by the nuclear accident in 2011. Meanwhile, quantitative surveys in 2013 showed that the number of species and population densities in the intertidal zones were much lower at sites near or within several kilometers south of FDNPP than at other sites, and lower than in 1995, especially in the case of Arthropoda. Although there was no clear explanation for these findings, it was evident that the intertidal biota around FDNPP had been affected since the nuclear accident [3].

Following the survey in 2013, Horiguchi et al. [4] conducted quantitative quadrat surveys on sessile invertebrates at seven intertidal sites in Ibaraki, Fukushima, and Miyagi Prefectures, including sites near FDNPP, in June 2014, May and June 2015, and June 2016, to check whether the number of species, population densities, and biomass had recovered from the decline after the 2011 disaster. Additionally, in April, July, August and September from 2012 to 2017, they monitored the population density and spawning behavior of the rock shell (*R. clavigera*) at sites near FDNPP. No increases in the number of species and population densities in the intertidal zone near FDNPP were found until at least 4–5 years after the FDNPP accident. Densities and reproductive performance of *R. clavigera* populations near FDNPP in 2017 remained at low levels. Although it was reasonable to expect invertebrate larval recruitment from remote areas to the intertidal zone near FDNPP, this was not clearly observed until 2016 at the earliest. Thus, Horiguchi et al. [4] suggested that it was possible that environmental factors had inhibited invertebrate reproduction, recruitment, or both, in the intertidal zone near FDNPP for at least 5 years.

Although no *R. clavigera* specimens were found near the plant from Hirono to Futaba Beach (a distance of approximately 30 km) in April 2012 [3], rock shells were again found to inhabit the area, such as at Okuma, located at approximately 1 km south of FDNPP in July 2016. Horiguchi et al. [5] then collected rock shell specimens monthly at two sites near FDNPP (Okuma and Tomioka, Japan) and at a reference site approximately 120 km south of FDNPP (Hiraiso, Japan) from April 2017 to May 2019. They examined the gonads of the specimens histologically to evaluate their reproductive cycle and sexual maturity. The gonads of rock shell collected at Okuma exhibited continuous sexual maturation during the two years from April 2017 to May 2019, whereas specimens from Hiraiso showed sexual maturation only in summer. The continuous sexual maturation observed at Okuma might not represent a temporary phenomenon, but rather a site-specific phenotype, possibly caused by specific environmental factors near FDNPP.

Kodama et al. [6] conducted bottom-trawl surveys independently of fisheries along the coast of Fukushima, Japan, from 2013 to 2017 to study the megabenthic community structure after the 2011 disaster. They observed no substantial changes in biodiversity, total abundance and biomass, which fluctuated among the years, primarily due to temporary increases in the abundance or biomass of small shrimp and squid, or variations in the abundance or biomass of mid-sized fishes (i.e., puffers and flatfishes) and large elasmobranchs. However, echinoderm abundance and biomass decreased in all areas. Moreover, crustacean abundance and biomass were extremely low in the central and southern offshore transects. Kodama et al. [6] suggested that there had been no recognizable recovery in the megabenthic community off the coast of Fukushima, and that megabenthic species off the coast of Fukushima might have been experiencing reproductive or recruitment failure.

Unfortunately, there are no data collected immediately after the disaster to support or reject possible reproductive or recruitment failures in the megabenthic community in the coastal waters off Fukushima. Therefore, discussions about possible causal factors for these phenomena must be speculative. Further research is needed to reveal the causal factors behind changes in these megabenthic communities. Here, to consider causal factors for the phenomena observed in invertebrates in the intertidal zone near FDNPP, we review the effects of ionizing radiation on aquatic organisms and their populations and ecosystems to discuss them in the context of the 2011 disaster.

## 2. Effects of Radionuclides and Ionizing Radiation on Aquatic Organisms

There have been many reports on the effects of ionizing radiation or radionuclides, such as ^90^Sr, ^137^Cs and ^3^H, on aquatic organisms. The ranges of acute lethal radiation doses for adults of various groups of organisms and experimental results for the effects of radionuclides in water on aquatic organisms are summarized in Table 1 and Table 2, respectively [7,8].

There have been several reports on the concentrations of radionuclides such as ^131^I, ^137^Cs and ^90^Sr in the coastal waters of Fukushima [43,44,45]. Concentrations of ^131^I and ^137^Cs in surface seawater in the vicinity of FDNPP in late March to early April 2011 were over 10^5^ Bq/L and approximately 10^5^ Bq/L, respectively [45]. Based on the ^137^Cs:^90^Sr ratio, the maximum concentration of ^90^Sr would have been around 10^4^ Bq/L [46]. Unfortunately, there is less information about the concentrations of radionuclides other than ^131^I and ^137^Cs in seawater, which leaked from the reactors into the sea. It may be useful to compare the total amount of ^137^Cs emitted from the Windscale nuclear units (currently the Sellafield Thermal Oxide Reprocessing Plants, located in northwestern England) to the sea. That is considered to be 41 PBq from 1952 to 1992, and the maximum annual emission of ^137^Cs was 5.2 PBq in 1975 [47]. The Fukushima nuclear disaster released almost the same amount of ^137^Cs (5.5–5.9 PBq) into the coastal waters of Fukushima as the maximum annual emission from the Sellafield plants over a relatively short period (from mid-March to early May 2011) [48], suggesting that the marine organisms around FDNPP might have experienced acute or sub-acute, rather than chronic, exposure to ^137^Cs and other radionuclides.

In discussing the effects of radionuclides/ionizing radiation on organisms, it is important to recognize that the degree of damage depends on the absorbed radiation dose, although the interactions between the physiological and pharmaceutical effects of their chemical forms should also be considered [7]. Wildlife, especially invertebrates, is tolerant of gamma radiation, although some mortality is expected in larvae and hatchlings of flatfish at 100–1000 mGy/d. Flatfish show reduced reproductive success at 10–100 mGy/d and reduced reproductive success due to reduced fertility is possible at 1–10 mGy/d. Invertebrates, such as crustaceans and mollusks, are more tolerant of radiation than are flatfish [49]. Acute lethal doses (LD_50_) for marine invertebrates, fish, and fish embryos have been estimated at >100 Gy, 10–25 Gy and 0.16 Gy, respectively [50]. Chronic exposure has yielded no-observable-effect dose rates of 10–30 mGy/h for mortality and 3.2–17 mGy/h for reproductive capacity in snails, scallops, clams and crabs. The no-observable-effect dose rate for reproduction in fish is 1 mGy/h [51,52,53]. Overall consideration of the data available led to the conclusion that maximum dose rates of less than 400 μGy/h to any individual in aquatic populations of organisms would be unlikely to have any detrimental effects at the population level [51,52,53].

We should also note that the effect of an absorbed dose depends on the type and energy of the irradiation. The type of irradiation and its energy are taken into account in radiation biology by using a relative biological effectiveness (RBE) factor that normalizes the ability of different radiation types to produce the same biological effect [54]. The value of the RBE of a test radiation is conventionally determined against a known standard radiation for a chosen response of selected biological tissue, and is expressed as the ratio of doses absorbed by tissue at equal effect, or as the ratio of magnitudes of the effect at equal absorbed dose [55]. Future studies should consider the RBE to determine impacts on intertidal invertebrates near FDNPP immediately after the accident, which would help us understand the mechanisms of their defaunation in its vicinity.

In another approach, the International Commission on Radiological Protection (ICRP) designated 12 general types of Reference Animals and Plants (RAPs) and summarized the dose rates that possibly cause them adverse effects [49]. The ICRP RAPs were chosen using a variety of taxonomic and practical criteria to serve as points of reference in ecological risk assessments [56]. The radiosensitivity of each reference organism has been documented in the literature, regarding four individual organism-level endpoints (i.e., mortality, morbidity, reproductive success, and mutation frequency). This method, based on traditional toxicology, emphasizes individual organisms rather than populations or ecosystems [56].

Although crustaceans (i.e., “crab”) are represented in the list of RAPs, mollusks are not [49]. Additionally, this “crab” is not supposed to be an intertidal species, but rather a demersal one, with 400–4000 μGy/h as the Derived Consideration Reference Level (DCRL) range [49]. Values of DCRL are intended to serve as points of reference in assessing the potential effects of ionizing radiation on non-human biota. A DCRL can be considered as a band of dose rates within which there is likely to be some chance of deleterious effects from ionizing radiation on individuals of that type of reference animal or plant. It is not clear, however, how effects at the individual level can be extrapolated to effects at population levels, and DCRLs only refer to individuals. Although intertidal invertebrates, such as gastropods (mollusk) and barnacles (crustacean) are not included in the list of RAPs, the ebb and flow of the tide in the intertidal zone could result in changes in exposure to radionuclides/ionizing radiation even within a single day. Therefore, it is necessary to carry out further research on dose estimations for intertidal invertebrates.

A Task Group of the International Union of Radioecology has presented the rationale to add an ecosystem approach for the tools available to manage radiation safety [57]. Bradshaw et al. [56] claim a broad consensus that environmental protection is better served by methods and concepts targeting populations and their interactions with other biota and abiotic components of ecosystems, compared to the ICRP RAPs, where the relationships between individual-level responses and population-level impacts of any kind of disturbance are tenuous [56]. Interactions among species, as well as life-history traits, physiological characteristics, and tolerances, could be more important for determining interspecies differences in susceptibility to radiation than differences in radionuclide-specific dose responses [56]. This rationale suggests that ecological knowledge is essential to better understand the responses of populations to radiation [56].

## 3. The Gap between Effects Observed in the Laboratory and Those in Aquatic Environments

On the basis of existing knowledge, as described above, and information about radionuclide concentrations in surface seawater around FDNPP in late March and early April 2011 [46], it would seem unlikely that there would be mass mortality (including the subsequent reproductive and/or recruitment failure) in intertidal invertebrates near FDNPP after the accident. What, then, caused a possible mass mortality event in intertidal invertebrates near FDNPP after the accident?

A report by Kosheleva [20] is worth our attention: mortality was observed during the entire embryonic development of the Atlantic salmon (*Salmo salar*), with high mortality when eggs had been exposed to a mixture of nuclear fission products at 1 × 10^−10^ Ci/L (=3.7 Bq/L). High mortality of eggs was also observed in water containing ^137^Cs at 1 × 10^−8^ Ci/L (370 Bq/L). Various radionuclides might be included in a mixture of nuclear fission products. Therefore, future studies should examine the effects from mixtures of radionuclides (including short-lived ones) to evaluate their impacts (i.e., impacts via internal as well as external exposure) on intertidal invertebrates near FDNPP after the accident. A variety of radionuclides were emitted into the atmosphere following the accident, and transport model simulations have indicated that more than 80% of the atmospheric fallout during the 2011 disaster was onto the ocean surface, and that deposition was highest onto coastal waters near FDNPP. In addition, contaminated material, including radionuclides, leaked directly from the reactors into the sea during emergency cooling efforts [58,59].

Differences in sensitivity to ionizing radiation among species should be also taken into consideration. Although genetic and other effects of ionizing radiation from acute/chronic exposure have been investigated, the test species for aquatic organisms are almost all small fishes, which are easy to maintain in the laboratory [7,8]. A limited number of other species, such as Artemia and Daphnia, have also been used as test species among invertebrates [7,8]. Thus, a site-specific species, such as the rock shell, should be used as a representative invertebrate for the intertidal zone near FDNPP in future laboratory exposure experiments, focusing on metrics such as lethality, growth, sexual maturation, spawning, and hatching. Ideally, the test should involve various developmental stages and early life-history stages of the species to observe their sensitivities to ionizing radiation.

Furthermore, it is possible that the field observations by Horiguchi et al. [3] (a decrease in the number of intertidal species and no rock shell specimens near the plant in 2012) suggest that mass mortality (including subsequent reproductive and/or recruitment failure) actually occurred in intertidal invertebrates near FDNPP after the accident. Although the Tokyo Electric Power Company began measuring concentrations of radionuclides in the surface seawater near FDNPP after the accident from 21 March 2011, there are no data for the concentrations within the 10 days from 11 to 20 March in the immediate aftermath of the accident [3]. It is therefore possible that higher concentrations of radionuclides would have detected in the surface seawater near FDNPP had such analyses been conducted during this period. Conversely, assuming that field observations by Horiguchi et al. [3] indicate mass mortality and reproductive and/or recruitment failure in intertidal invertebrates near FDNPP after the accident, then the total amount of radionuclides emitted from FDNPP to the environment might have been underestimated.

Clearly, there is a considerable gap between the effects observed in aquatic organisms in laboratory experiments and those observed in a contaminated field site, such as the intertidal zone near FDNPP. It is necessary to clarify the reasons for this gap in the future.

Assessing the effects of ionizing radiation on aquatic populations is necessary and useful for the protection of aquatic ecosystems from the risk of contamination by radionuclides or exposure to ionizing radiation resulting from nuclear accidents. Actually, however, it is difficult to assess the effects of ionizing radiation on aquatic populations. First, as mentioned above, there is a bias of test species used in laboratory experiments (i.e., small fishes in many cases and a limited number of invertebrate species, such as Artemia and Daphnia, most of which are easy to maintain in the laboratory). Considering the differences in sensitivity to ionizing radiation among species, test species should include a site-specific species inhabiting the intertidal zone near FDNPP—such as the rock shell—species that might have more important ecological roles, and those that are important target species for commercial fisheries, for a more realistic evaluation of the effects of ionizing radiation, although these species may be more difficult to culture and maintain in the laboratory. Some of these species may be more sensitive to ionizing radiation than the test species used in typical laboratory experiments. Second, the concentrations of radionuclides and levels of ionizing radiation to which test species are exposed in the laboratory seem much higher than those detected in the natural aquatic environment, even at many contaminated sites in Fukushima. It could be difficult to extrapolate the effects observed in aquatic organisms exposed to relatively high doses or dose rates in the laboratory (i.e., hundreds or thousands of rad [=several or several tens of Gy]) to ecologically relevant effects observed in aquatic organisms in the field, which could be chronically exposed at much lower doses/dose rates.

One topic that has long been of concern is whether exposure to ionizing radiation has any adverse effects on aquatic organisms at population, community, and/or ecosystem levels. Even at the contamination levels from artificial radionuclides in the 1960s and 1970s due to atmospheric nuclear weapon testing, effects were considered to be absent or minor at the population level both for species with high fecundity (i.e., teleost fishes) and for those with low fecundity (i.e., elasmobranchs and marine mammals) [8]. Results from field surveys/experiments (including at the Chernobyl Evacuated Zone [CEZ]) using fish populations for long-term (or chronic) exposure to ionizing radiation at low doses/dose rates suggest that there are no effects at population levels even if there are inhibitory effects on maturation in gonads of adults, and effects on hatching, deformity and survival rates in the early life-history stages at the individual level [60]. Possible effects from ionizing radiation at low doses/dose rates in the field may also be obscured by those from other environmental factors as well as by natural fluctuations of abundance in the population (including a density-dependent response) [8]. It will be necessary to assess the effects of long-term (chronic) exposure to radiation at low doses/dose rates and the combined effects of ionizing radiation with other environmental factors to evaluate genetic damage as well as population-level effects [8].

**Figure 1:**
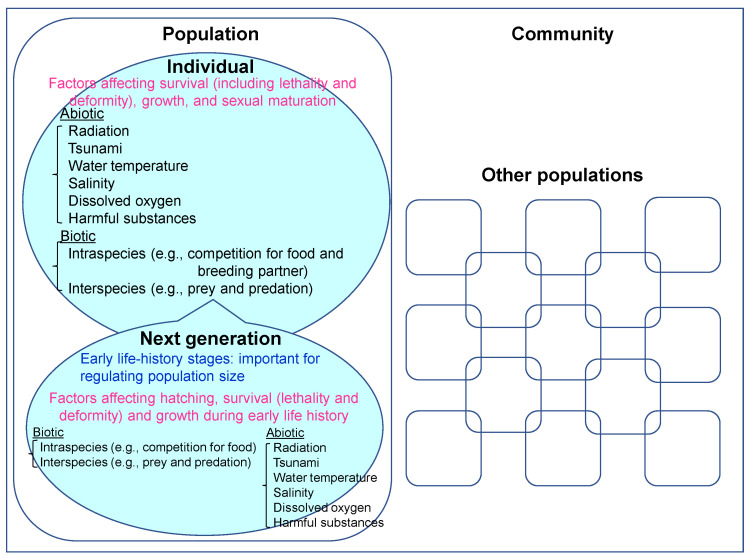
Biotic and abiotic factors affecting the survival (including lethality and deformity), growth, and sexual maturation of each individual in a population, and of subsequent generations.

In the natural environment, several biotic/abiotic factors affect the survival (including lethality and deformity), growth and sexual maturation of each member of a population (Figure 1). It is especially important to consider the effects on hatching, survival (lethality and deformity) and growth during the early life-history stages to evaluate population-level effects, because mortality/survival rates in the early life-history stages could regulate the population size. Therefore, we also need to focus on biotic and abiotic factors affecting hatching, survival (lethality and deformity) and growth during early life history to consider the population-level effects from ionizing radiation (Figure 1).

Regarding impacts of the 2011 Tohoku Earthquake Tsunami on intertidal organisms, several papers and books have been published. For example, Urabe et al. [61] investigated changes in taxonomic composition and richness of macrobenthic animals at nine intertidal flats in Sendai Bay and the Sanriku Ria coast, Japan, to assess the immediate impacts of the tsunami on coastal communities. The results showed 30–80% of taxa indigenously inhabiting intertidal flats disappeared after the tsunami. Among animal types, endobenthic and sessile epibenthic animals were more vulnerable to the tsunami than mobile epibenthic animals, such as shore crabs and snails. For all the intertidal flats examined, animals that were originally dwellers in lower tidal zones and not recorded before the tsunami were also found immediately after the tsunami, indicating that the tsunami not only took away many benthic taxa from the intertidal flats but also brought in some taxa from elsewhere. However, overall changes in taxonomic composition in the communities were greater for intertidal flats that experienced larger inundation heights. It is suggested that the ecological impacts of the tsunami were proportional to the physical impacts as gauged by wave height and that mobile epibenthic animals were less vulnerable to the tsunami [61]. Noda et al. [62] examined the vertical distribution of 10 dominant macrobenthic species (six sessile and four mobile species) in the mid-shore zone of 23 sites along the Sanriku coastline, Japan, 150–160 km north-northwest of the earthquake epicenter, and compared the vertical distributions of each species with their vertical distributions in the pre-earthquake period. They found that dynamics of rocky intertidal zonation varied substantially among species. Among sessile species, one barnacle dramatically increased in abundance and expanded its vertical range in 2011, but then decreased and completely disappeared from all plots by 2013. Zonation of other sessile species shifted downward following the subsidence in 2011. With some species, there was no clear change in abundance immediately after the earthquake, but they began to increase and move upward after a few years. Abundance continuously decreased in other sessile species. Meanwhile, there was no clear change in the vertical distribution of any of the mobile species immediately after the earthquake. Abundance of two mobile species was unchanged, but abundance of the others decreased from 2012 and had not recovered as of 2013 [62].

## 4. Effects of Biotic and Abiotic Factors Other than Ionizing Radiation on Aquatic Organisms

There are many reviews as well as original papers on the effects of biotic and abiotic factors other than ionizing radiation on aquatic organisms at population levels (for example, Buhay [63], Kodama and Horiguchi [64], Trowbridge et al. [65]). Biotic factors include those such as prey and predation, and abiotic factors are such things as destruction/loss of habitat due to reclamation of tidal flats and shallow waters, changes in water temperature and dissolved oxygen (i.e., hypoxia), and harmful substances, such as heavy metals and chemical substances. Here, we show several case studies [66,67,68,69].

Lee et al. [66] investigated factors that might have disturbed the stock recovery of the marbled flounder in Tokyo Bay, Japan, from 2006–2011 by focusing on its early life stages. Field surveys from 2006 to 2011 revealed that mature adult biomass increased from 2006 to 2008 and decreased thereafter. In the meantime, larval and juvenile densities were high in 2006 and 2008, but low in other years. Variations in the yearly trends of these parameters imply that mortality during life stages between spawning and early larval phases might have affected the abundance of the subsequent life stages. Monthly mean water temperature in January and February, during which hatching occurs and pelagic larvae are observed in the bay, was lower in 2006 (8.6 °C) and 2008 (9.6 °C) than in other years (10.4–11.4 °C). The significant negative correlation between water temperature and larval density suggests that mortality during pre- and post-larval stages would be higher in years with warmer winters (>10 °C). To test this hypothesis, they examined the effects of water temperature on mortality and development in eggs and larval stages under controlled laboratory conditions. The hatching rate was higher in water temperatures of 9.2–12.7 °C (66.6–82.5%), whereas it decreased in cooler (3.7% at 5.9 °C) and warmer (33.9% at 14.8 °C) conditions. On the other hand, the number of days from fertilization to hatching, size of larvae at hatching, and survival of larvae after 18 d from hatching monotonically and significantly decreased as water temperature increased. The combined evidence from the field and laboratory studies implies that a warmer reproductive season (>10 °C) might induce higher mortalities in the larval stages of marbled flounder in Tokyo Bay.

Kodama et al. [67] investigated the effects of severe hypoxia (dissolved oxygen < 1 mL/L) on recruitment of the mantis shrimp *Oratosquilla oratoria* in Tokyo Bay. Ten-year field surveys were carried out to examine quantitative relationships between annual mean densities of larvae and juveniles, and between spatial distributions of juveniles and severe hypoxia. There was no significant correlation between annual mean densities of larvae and juveniles, implying that mortality during larval or juvenile stages varies among years, which might regulate the abundance of young-of-the-year juveniles. Juvenile density was low in areas with severe hypoxia, suggesting that hypoxia could influence the survival and spatial distribution of juveniles. There were also yearly fluctuations in juvenile densities in normally oxic areas of both the northern and southern parts of the bay. This evidence implies that the abundance of post-settled juveniles might be determined not only by the effects of hypoxia, but also by other factors affecting mortality during early life stages.

Horiguchi et al. [69] performed histopathological examination of gonads and measured organotin compounds, such as tributyltin (TBT), triphenyltin (TPhT) and their metabolites, in tissues of the ivory shell *Babylonia japonica*. Imposex—the superimposition of male-type genital organs (i.e., the penis and vas deferens) on females, induced by TBT and TPhT from antifouling paints—occurred in approximately 80–90% of *B. japonica* specimens that they examined. They observed no oviduct blockage by the formation of vas deferens, suggesting no physical obstruction for spawning egg capsules. However, they did observe ovarian spermatogenesis and suppressed ovarian maturation in the females that exhibited imposex, although no histopathological abnormalities were found in males. The distributions of TBT, TPhT, and their metabolites in tissues were different for butyltins and phenyltins: a remarkably high accumulation of TBT was observed in the ctenidium, osphradium, and heart. Meanwhile, high concentrations of TPhT were detected in the ovary and digestive gland. Both TBT and TPhT concentrations in the gonads were positively correlated with penis length in females. Their findings strongly suggest that reproductive failure in adult females accompanied by imposex, possibly induced by TBT and TPhT from antifouling paints, might have caused the marked decline of *B. japonica* populations in Japan. Regarding this population decline, however, Horiguchi et al. [69] also pointed out that early life-history characteristics were critical. Because the planktonic stage of *B. japonica* is estimated to last approximately 4–5 days [70,71], the recruitment of veliger larvae from other populations inhabiting remote, less contaminated areas is unlikely. Reproductive failure accompanied by imposex in female ivory shell could result in extirpation of a *B. japonica* population within several years because the number of offspring produced by adults is likely to continue to decrease. The existence and duration of a free-swimming phase during larval development is one of the important factors in determining the linkage between impaired reproductive ability from imposex and population decline.

Kodama et al. [68] indicated changes in the growth and reproductive traits of the dragonet *Callionymus valenciennei* in Tokyo Bay that coincided with a decrease in stock size. Changes in life-history traits and population dynamics of marine organisms in response to environmental change are known to occur in nature, although the causes underlying these responses and the mechanisms remain unidentified [72]. The minimum standard length at which the dragonet attains gonadal maturation was smaller in the 2000s (4.8 cm) than in the 1990s (6.0 cm). Additionally, the timing of the onset of spawning was earlier in the 2000s (spring) than in the 1990s (summer). Kodama et al. [68] also found out significant changes in growth in both sexes from the 1990s to the 2000s; growth after sexual maturity was significantly lower in the 2000s compared with the 1990s. Such changes in life-history traits of the dragonet might reflect a trade-off in the allocation of available energy resources between reproduction and somatic growth; under limited prey abundance, more energy resources might be allocated to reproduction to enhance stock recovery in Tokyo Bay.

## 5. Existing Knowledge on Combined Effects of Ionizing Radiation and Other Environmental Factors

There have been several reports on how environmental factors influence the effects of radiation. In the goldfish *Carassius auratus*, most fish survived for more than 100 days when they were kept at 4 °C after they had been exposed to 8 kR (8000 rad, 80 Gy) whole-body irradiation; meanwhile, most similarly irradiated fish died within 10 days at 22 °C. Low temperature inhibited the development of damage in the intestinal epithelium. Those irradiated fish that were maintained at 4 °C showed marked histological damage of the intestinal epithelium at more than 100 days after irradiation (approximately 75% of the fish died between 150 and 200 days after irradiation). Irradiated fish maintained at 4 °C for various durations and then transferred to 22 °C died within about 10 days, exhibiting similar damage of the intestinal epithelium [73,74]. Meanwhile, the hatchability of irradiated dry eggs of *Artemia salina* was temperature-dependent [75]. Irradiated dry eggs kept at dry-ice temperature did not show any decrease in hatchability, which could be interpreted as resulting from low temperature preventing the amplification of the damage due to the “storage effect” [76].

Angelobic et al. [77] observed that estuarine species, the mummichog *Fundulus heteroclitus* and the grass shrimp *Palaemonetes pugio* were more resistant to radiation at lower salinities. Angelobic et al. [78] reported on the combined effects of ionizing radiation, salinity and temperature on the mummichog. In a factorial experiment, mummichogs were subjected to four levels of acute radiation (500, 1000, 2000 and 2500 rad [=5, 10, 20 and 25 Gy]), three salinities (5, 15 and 25 [‰]), and four temperatures (12, 17, 22 and 27 °C). They found that different combinations of temperature and salinity yielded different LD_50_ values. The estimated LD_50_ for different experimental conditions ranged from 300 to 350 rad (3 to 3.5 Gy) to more than 2500 rad (25 Gy). The mummichog tolerated more radiation at low salinity at the upper end of the temperature range. At the lower end of the temperature range, tolerance was reversed. Because irradiated fish generally lose sodium more rapidly than unirradiated fish, it can be assumed that some of the lethal effects of radiation would be ascribed to damage to osmoregulatory capabilities. This seemed true in the case of irradiated salmon *Oncorhynchus kisutch* that were exposed to 1000 rad (10 Gy) or more and then transferred from fresh to saline water [79]. Approximately 50% of the transferred salmon died within 60 days, whereas the salmon remaining in freshwater (and therefore not subjected to osmotic stress) were unaffected [79].

These studies all suggest that a more realistic evaluation of the effects of ionizing radiation on aquatic organisms inhabiting natural/contaminated environments should be assessed with consideration for the effects from other environmental factors as well.

## 6. The Environment Is a Complex System: The Necessity of Evaluating Combined Effects of Ionizing Radiation and Other Biotic and Abiotic Environmental Factors for Population-Level Assessments

Recognizing that the environment is a complex system, we note that it is essential to evaluate interactions between ionizing radiation and other environmental (abiotic and biotic) factors for the realistic assessment of population-level effects, taking into consideration site-specific characteristics of the species, such as reproductive season, breeding style, ecology during early life history, prey and predators, and hydrographic conditions. To properly understand the effects of radionuclide exposure on organisms in the context of a nuclear accident, it is also essential to distinguish at least two contrasting phases in terms of the level and type of exposure: an acute exposure phase, with a higher dose lasting a few weeks (up to 2–3 months) immediately after the accident, and a chronic exposure phase, with a lower dose lasting many years [80].

The acute phase, extending the initial weeks (up to 2–3 months) following an accident, is characterized by the presence of a large quantity of short-lived radionuclides likely to expose organisms to high dose rates, primarily through external irradiation, with a significant proportion of the dose delivered by beta-emitting radionuclides [80]. During this phase, acute effects are likely to be observed [80]. Acute effects include any notable biological modification occurring within a few days or weeks of absorption of a significant radiological dose, leading to irreversible damage and, eventually, death [80]. The later or chronic phase takes place on a scale of several months to years, during which the contamination levels in the environment change much more slowly [80]. The effects are of many types, but come with the large uncertainties inherent in the extrapolations required to interpret any ecological significance of the effects observed at different levels of biological organization [80].

In addition to evaluating dose rates and effects at the individual level, Bréchignac et al. [81] pointed out the importance of assessing the impacts of radiation at population, community, and ecosystem levels. From this perspective, they enumerated three research priorities: systems-level research, additional research at the organism level, and field studies to calibrate the results from laboratory studies at both the systems and organism levels.

Considering the effects from ionizing radiation and the subsequent recovery process in certain organisms at population levels, the phenomena observed in a contaminated field situation is complicated rather than simple. For example, the acute impact of high doses of radiation was reportedly the clear eradication of some local populations in the CEZ, but those populations were eventually restored through immigration [60]. Apparently, this is similar to the case of the disappearance of rock shell (*R. clavigera*) populations near FDNPP in 2012 and their subsequent recovery, which was delayed until 2016 [4]. No rock shell specimens were collected at eight sites near FDNPP in Fukushima Prefecture in 2012 [3]. However, because rock shell specimens were collected in 2012 at many sites in Miyagi and Iwate Prefectures, as well as at sites in northern Fukushima Prefecture, where the tsunami also hit, it is unlikely that the absence of rock shell near FDNPP resulted only from the tsunami [3]. The absence of rock shell at sites around FDNPP (from Hirono to Futaba Beach, a distance of about 30 km) also implies that reproduction and recruitment did not occur there, or were less successful, in summer and autumn (the reproductive season and thereafter) in 2011 [3]. This is in addition to the possible mortality of almost all individuals living there after March 2011, although it is still necessary to elucidate why adult rock shells living there disappeared, or why rock shells had little or no reproductive success there [3]. The early life history of the rock shell includes a free-swimming planktonic veliger stage that lasts for more than 2 months [82]. Therefore, larval recruitment from remote areas to the intertidal zone near FDNPP was expected. In reality, there was a gradual redistribution of the rock shell from northern and southern sites to the sites near FDNPP in the central area of Fukushima Prefecture from 2012 to 2016 [4]. Despite the recovery of the rock shell populations near FDNPP, however, continuous sexual maturation, a strange, abnormal phenomenon, has been observed in rock shell collected at Okuma, ~1 km south of FDNPP, since April 2017 [5].

To summarize, we believe that the reasons for the gap between the effects of ionizing radiation observed in the laboratory and those in actual aquatic environments (i.e., intertidal zones near FDNPP) are as follows:

(1) Limitations on extrapolation from the effects observed in aquatic organisms exposed at relatively high doses/dose rates in the laboratory to ecologically relevant effects observed in aquatic organisms in the field, which could be exposed chronically at much lower doses/dose rates.
(2) Insufficient consideration of the combined effects of ionizing radiation with other abiotic and biotic environmental factors together with the life-history traits of the species examined, for assessment of population-level effects.
(3) The misapprehension that population-level effects can be assessed by simply extending the individual-level effects on adults to the next generation.

We have already discussed how it can be difficult to extrapolate from the effects observed in aquatic organisms exposed at high doses/dose rates in the laboratory (i.e., hundreds or thousands of rad = several or several tens of Gy) to ecologically relevant effects observed in aquatic organisms in the field, which could be exposed chronically at much lower doses/dose rates. This is because the concentrations of radionuclides or levels of ionizing radiation to which test species are exposed in the laboratory seem much higher than those detected in the natural aquatic environment, even at many contaminated sites in Fukushima. Although we also think that it is important to investigate the effects of long-term (chronic) exposure to radiation at low doses/dose rates, we also need to recognize that the environment is a complex system (Figure 1). It is therefore essential to evaluate the combined effects of ionizing radiation and other environmental factors for the realistic assessment of population-level effects. Decreases in gametogenesis in adults (i.e., inhibition of sexual maturation of gonads) by exposure to radiation may not necessarily result in a smaller population because of compensative recruitment of larvae from remote areas. It is known that a population with high abundance may become a population with low abundance within a few generations, and vice versa, due to changes in mortality or survival rates in the early life-history stages. Consideration of the effects on hatching, survival (lethality and deformity), and growth, in the early life-history stages, is critical to evaluating population level effects, because it is known that the mortality/survival rate in the early life-history stages can regulate the size of a population (Figure 1). Therefore, it is necessary to assess the combined effects of ionizing radiation and other environmental factors for both adults and early life-history stages, separately or independently, focusing on ecologically relevant endpoints (e.g., survival of each individual in a healthy condition, reproductive success in terms of sexual maturation, copulation, spawning and recruitment) to maintain the population in a contaminated site.

We therefore present the following possible causes for the strange/abnormal phenomena observed in invertebrate populations in intertidal zones near FDNPP:

(1) **Decline in population densities as well as the number of species.** Radionuclides (including short- and long-lived ones) are candidates as causal factors, as discussed by Horiguchi et al. [3]. Even if there was no mass mortality in intertidal invertebrate populations near FDNPP immediately after the nuclear accident, it is possible that long-term survival of individuals exposed to radiation might have been difficult with the possible depletion of prey and the unchanging, harsh intertidal environment along the coast of Fukushima. Although boric acid and hydrazine, which were also thought to have been released from FDNPP into the sea at the time of the accident, might also be causal factors, we believe it unlikely that they would have caused mass mortality in intertidal invertebrate populations near FDNPP after the nuclear accident, because seawater already contains much boron, and hydrazine decomposes rapidly in sunlight.
(2) **Delayed recovery from the decline in population densities and the number of species.** Several heavy metals and a possible increase in pH resulting from the cement used in the construction to cover the contaminated bottom sediment in the harbor at FDNPP may be causal factors reducing the survival rates of invertebrate larvae recruiting from remote areas to the intertidal zone near FDNPP [4]. The possible increase in turbidity due to the construction may also be a factor [4].
(3) **Continuous sexual maturation in the rock shell population.** It is unlikely that radionuclides that leaked from FDNPP or high seawater temperatures are causal factors for these observed effects, as discussed in Horiguchi et al. [5]. However, it is possible that some unknown harmful substance(s) that may have leaked from FDNPP may be related to the induction/promotion and continued sexual maturation, or the failure to terminate maturation, in the rock shell population near FDNPP throughout the year. Unknown changes in physiological characteristics of the rock shell near FDNPP may be involved. There might also be a trade-off in the allocation of available energy resources between reproduction and somatic growth, because changes in life-history traits and population dynamics of marine organisms in response to environmental change are known to occur in nature, although the causes underlying such responses and the mechanisms remain unidentified [72].

## Figures and Tables

**Table 1:** Ranges of acute lethal radiation doses for adults of various taxonomic groups.

Group	(Krad)	(Gy)	Remarks ^#^
Bacteria	4.5–735	45–7350	LD_90_
Cyanobacteria	<400 to >1200	<4000 to >12000	LD_90_
Other algae	3–120	30–1200	LD_50_
Protozoa	<600	<6000	LD_50_
Mollusks	20–109	200–1090	LD_50/30_
Crustaceans	1.5–56.6	15–566	LD_50/30_
Fishes	1.1–5.6	11–56	LD_50/30_

This table is adapted from Table 33 in Ophel et al. [8]. Units of Gy have been added. ^#^ LD_50_ means a lethal dose for half (50%) of individuals tested. Similarly, LD_90_ means a lethal dose for 90% of individuals tested. LD_50/30_ represents a lethal dose for half (50%) of individuals tested within 30 days.

**Table 2:**
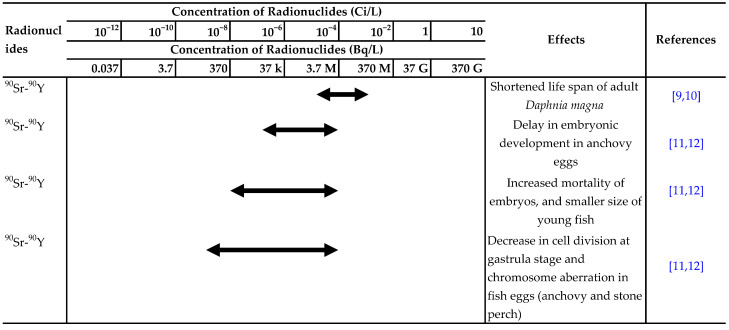

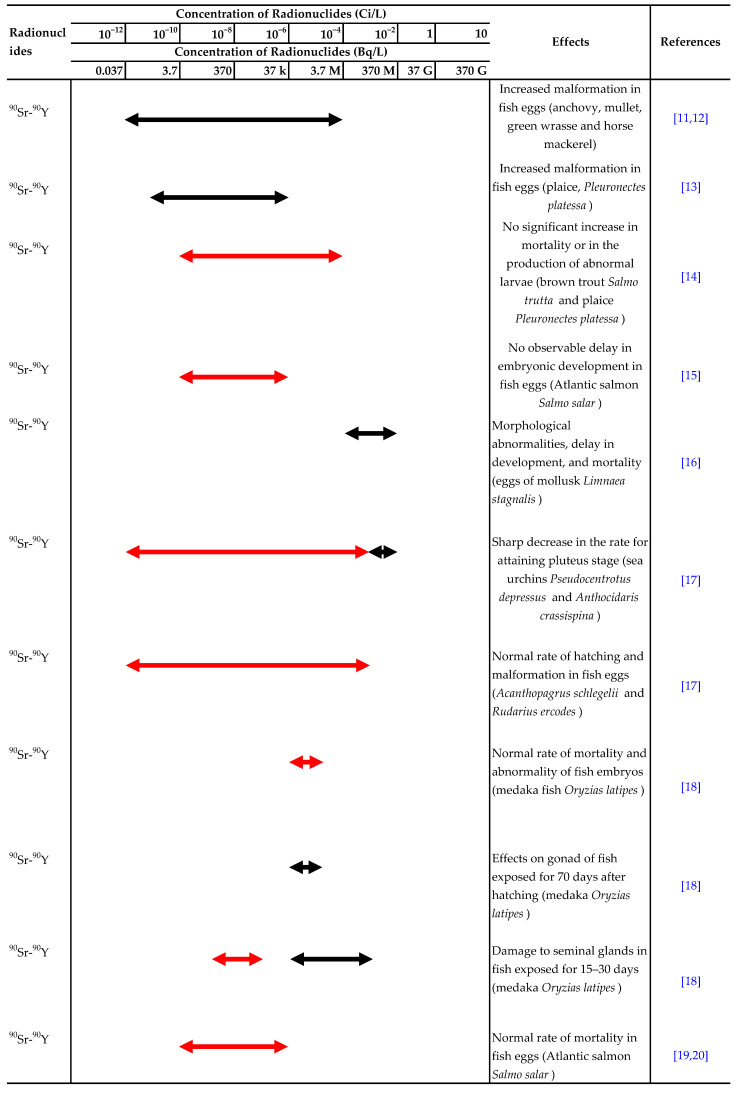

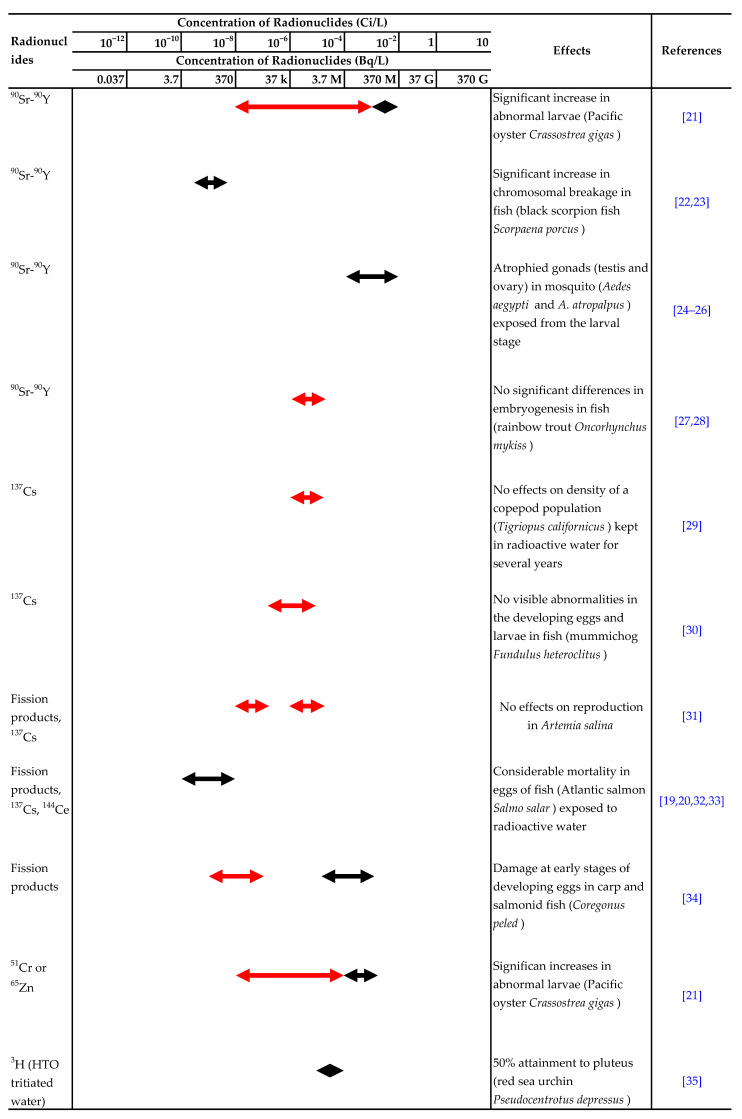

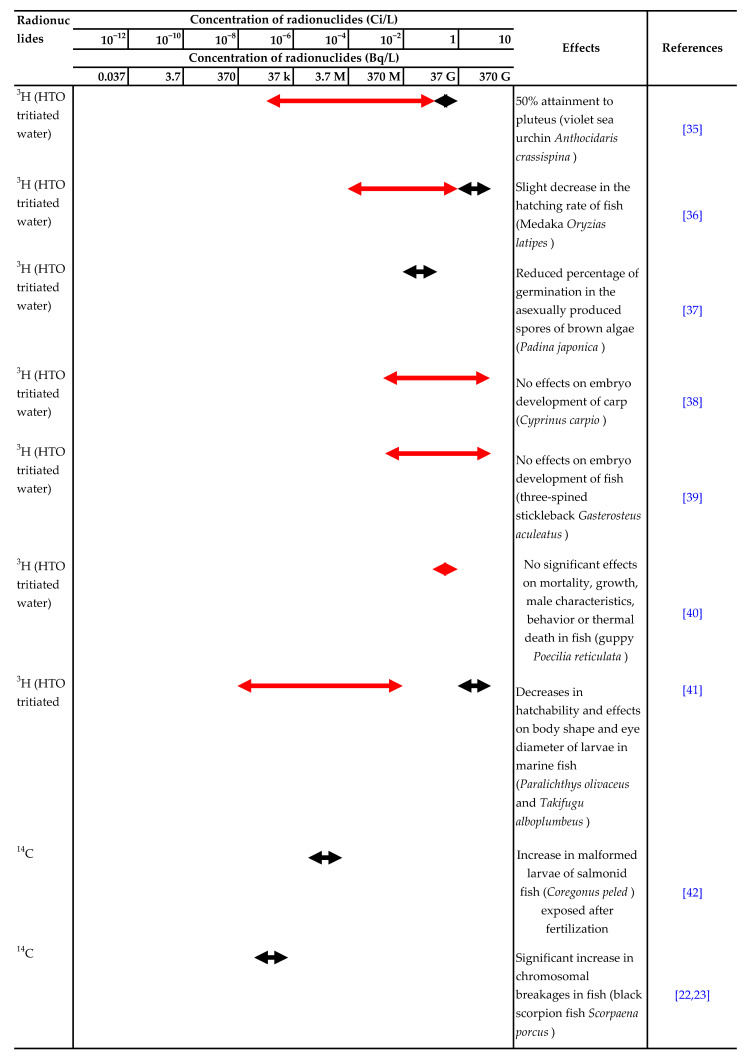
Summary of experimental results on the effects of radionuclides in water on aquatic organisms. Effect: 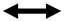 Detected. 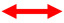 Not detected. Refs. [9,10,11,12,13,14,15,16,17,18,19,20,21,22,23,24,25,26,27,28,29,30,31,32,33,34,35,36,37,38,39,40,41,42] are cited in table.

This table is based on Table 35 in Ophel et al. [8] and Table 8.6 in Etoh [7]. Scientific names of several species have been corrected to those currently used. Units of Bq/L have been added. Note: The concentrations given do not directly indicate the doses received by organisms and/or organs.
